# Anti-Obesity Effects of Dietary Calcium: The Evidence and Possible Mechanisms

**DOI:** 10.3390/ijms20123072

**Published:** 2019-06-23

**Authors:** Fenglin Zhang, Jingjing Ye, Xiaotong Zhu, Lina Wang, Ping Gao, Gang Shu, Qingyan Jiang, Songbo Wang

**Affiliations:** 1 Guangdong Provincial Key Laboratory of Animal Nutrition Control, College of Animal Science, South China Agricultural University, Guangzhou 510642, China; zfl771896317@163.com (F.Z.); fengzhongyezi2009@163.com (J.Y.); xtzhu@scau.edu.cn (X.Z.); wanglina@scau.edu.cn (L.W.); gaoping@scau.edu.cn (P.G.); shugang@scau.edu.cn (G.S.); qyjiang@scau.edu.cn (Q.J.); 2 National Engineering Research Center for Breeding Swine Industry and ALLTECH-SCAU Animal Nutrition Control Research Alliance, South China Agricultural University, Guangzhou 510642, China

**Keywords:** anti-obesity, calcium, adipogenesis, fat metabolism, proliferation and apoptosis, thermogenesis, fecal fat excretion, gut microbiota

## Abstract

Obesity is a serious health challenge worldwide and is associated with various comorbidities, including dyslipidemia, type 2 diabetes, and cardiovascular disease. Developing effective strategies to prevent obesity is therefore of paramount importance. One potential strategy to reduce obesity is to consume calcium, which has been implicated to be involved in reducing body weight/fat. In this review, we compile the evidence for the anti-obesity roles of calcium in cells, animals, and humans. In addition, we summarize the possible anti-obesity mechanisms of calcium, including regulation of (a) adipogenesis, (b) fat metabolism, (c) adipocyte (precursor) proliferation and apoptosis, (d) thermogenesis, (e) fat absorption and excretion, and (f) gut microbiota. Although the exact anti-obesity roles of calcium in different subjects and how calcium induces the proposed anti-obesity mechanisms need to be further investigated, the current evidence demonstrates the anti-obesity effects of calcium and suggests the potential application of dietary calcium for prevention of obesity.

## 1. The Current Situation of Obesity and Its Adverse Effects

Obesity, defined as excessive fat deposition, has become increasingly prevalent worldwide. Over the last 40 years, the prevalence rates of obesity in adults (defined as BMI over 30 kg/m^2^) has been increasing at a rapid pace in both Western societies and developing countries, with the number of obese adults reaching 671 million in 2016 (390 million women and 281 million men) compared to 100 million in 1975 (69 million women and 31 million men) [1]. In addition, the prevalence of childhood obesity has increased steadily in the developed and developing countries [2]. For example, approximately one-third of children in America are overweight or obese [3,4]. A similar situation has occurred in China, with the prevalence of childhood overweight and obesity being about one in five [5,6].

Obesity has become a major public health burden all over the world. It is now well established that obesity is able to progressively lead to and/or exacerbate a wide range of comorbidities [7,8,9], including insulin resistance and type 2 diabetes mellitus (T2DM) [10,11], dyslipidemia [12,13], hypertension [14,15], cardiovascular disease [16,17], nonalcoholic fatty liver disease [18,19], reproductive dysfunction [20,21,22], and cancer [23,24]. As a result, obesity causes adverse effects on the quality of life and has marked economic consequences relating to increased healthcare costs [25,26]. In view of the prevalence of obesity, health consequences, and healthcare costs, there has been substantial interest in identifying effective and safe interventions/strategies to reduce excess body weight/fat in obese people.

Although there are many factors that influence obesity and various interventions to treat it [27], a large body of evidence has demonstrated that dietary interventions/strategies are an effective and safe way to prevent or manage obesity [28,29,30,31]. As one of the micronutrients in the diet, calcium regulates many cellular processes, such as cell proliferation [32], differentiation [33], and bone formation [34]. In addition, dietary calcium has been implicated to be involved in prevention or treatment of obesity [35,36,37,38,39,40]. Onakpoya et al. reported the efficacy of calcium supplementation for management of overweight people and aimed to clarify the treatment effect of calcium supplementation in obese people [36]. Soares et al. mainly demonstrated the effect of calcium and vitamin D on obesity [41]. Barba and Russo’s review focused on the association between dairy product consumption and body weight regulation in humans [40].These studies mainly focused on humans or the anti-obesity effect of calcium on overweight people. At present, there are few comprehensive reviews describing the anti-obesity effects of calcium in different models and the underlying mechanisms. In this review, we compile the evidence for the anti-obesity effects of calcium in cell models, animals, and humans and summarize the possible mechanisms by which calcium elicits its anti-obesity effects.

## 2. The Anti-Obesity Effects of Calcium Supplementation

### 2.1. Inhibition of Adipogenic Differentiation by Calcium in Cell Models

Expanded fat mass can result from increased adipocyte number (adipogenesis or hyperplasia) and/or increased adipocyte size (hypertrophy) [42]. A decrease in adipogenesis and lipogenesis and/or an increase in lipolysis contribute to fewer adipocyte number and smaller adipocyte size, thus leading to a reduction in fat accumulation. It has been reported that high extracellular calcium ([Ca^2+^]_o_, 5 and 10 mM) attenuates adipogenesis in 3T3-L1 preadipocytes [43]. Similarly, 5 mM [Ca^2+^]_o_ and increased intracellular calcium ([Ca^2+^]_i_) with RyR channel excitomotor caffeine significantly reduced the intracellular lipid content in the primary preadipocytes of mice [44,45]. In addition, increasing [Ca^2+^]_i_ was able to inhibit early stages of adipogenic differentiation in human preadipocytes [46]. Taken together, these in vitro data indicate that direct treatment of murine and human preadipocytes with calcium or calcium channel regulators could elicit inhibitory effects on adipogenic differentiation.

### 2.2. Anti-Obesity Effects of Dietary Calcium in Animals

Accumulating evidence has demonstrated that dietary calcium supplementation elicits anti-obesity effects on various animals. Our study has indicated that calcium supplementation (0.6% *w*/*w*) in drinking water leads to significant decrease in body weight, body fat content, and inguinal white adipose tissue (iWAT) and epididymal WAT (eWAT) index in high-fat diet (HFD)-induced obese mice [47]. In line with our report, Sun et al. found that dietary supplementation of 1.4% and 2.8% calcium significantly decreased the body weight gain and the fat net weight of inguinal fat pad (IFP), epididymal fat pad (EFP), and perirenal fat pad (PFP) in HFD-fed mice [45]. The anti-obesity or body-fat-lowering effects of calcium in mice have also been reported in other studies [48,49]. In rats, it has been shown that, compared with standard chow, calcium-supplemented chow (10 g CaCO_3_/kg of chow) significantly decreased body mass and visceral adipose tissue (VAT) mass in epididymal, retroperitoneal, and mesenteric depots [50]. In agreement, dietary calcium supplementation (10 g/kg) resulted in significant reduction of body mass, trunk fat, and total fat in early weaning Wistar rats [51]. With regard to the brown adipose tissue (BAT), it was reported that calcium-supplemented chow (10 g/kg) had no effects on rat BAT weight compared to standard chow [52]. Collectively, these data suggest that dietary calcium intake could elicit beneficial effects on reducing body fat deposition in murine models.

### 2.3. Anti-Obesity Effects of Dietary Calcium in Humans

Many studies have evaluated the effects of dietary calcium supplementation on body weight/fat loss in humans [38,39,53,54]. According to a survey, people in both developed and lesser developed countries have inadequate calcium intake [35]. The 2011 Institute of Medicine Dietary Reference Intake committee set the recommended dietary allowances at 1300 mg/day calcium for children aged 9–18 years and 1000–1200 mg/day (varying by age) for healthy adults [55]. In fact, most American children do not yet meet these recommendations [56,57]. Thus, increasing the intake of daily calcium is the primary condition for health and may contribute to body weight/fat loss. A meta-analysis revealed the negative correlations between calcium supplementation and weight changes in children and adolescents, in adult men, and either premenopausal or old (above 60 years old) women and suggested that increasing calcium intake could reduce body weight in these subjects [53]. Specifically, it has been demonstrated that each 300 mg increment in regular calcium intake is associated with approximately 1 kg less body fat in children and 2.5–3.0 kg lower body weight in adults [54]. Rosenblum et al. found that calcium and/or vitamin D supplementation contributed to a beneficial reduction of abdominal visceral adipose tissue in overweight and obese adults [58]. In contrast, Winzenberg et al. reported that there was no evidence to support the use of calcium supplementation as a public health intervention to reduce weight gain or body fat in healthy children [59]. It should be noted that vitamin D, which exerts a critical role in calcium absorption [60], plays an important role in influencing the anti-obesity effects of calcium. It has been reported that a deficiency of vitamin D decreases the calcium intake and increases body mass index in children and adolescents [61]. In addition, dietary calcium overdosage has been implicated in some adverse effects, including kidney stones, myocardial infarction, hypercalcemia, and hospitalization with acute gastrointestinal symptoms [62]. Excess (>1200 mg/day) dietary calcium intake is related to higher Framingham Risk Score (FRS), which is generally considered as a tool to assess future cardiovascular risk in humans [63].

The source of calcium may also affect its anti-obesity effects. It has been implicated that the anti-obesity role of calcium intake in children and adolescents might be driven exclusively by dairy calcium [64], implying that dairy calcium might be more effective than calcium supplements. Consumption of a high Ca diet from dairy for 12 weeks was effective in reducing abdominal adiposity in overweight patients with T2DM [65]. Greater intake of high-fat, but not intake of low-fat, dairy products, was found to be associated with less weight gain in middle-aged and elderly women [66]. It was also reported that increasing dairy calcium intake with low-fat milk or yogurt for 12 months had no effect on decreasing body fat or weight gain in overweight adolescent girls [67]. In addition, gender may also influence the anti-obesity effects on dietary calcium. Lee et al. found that consumption of dairy products is associated with reduced risks of obesity and metabolic syndrome in Korean women but not in men [68]. Similarly, Moreira et al. reported an inverse relationship between calcium intake and BMI in only girls (7–9 years old) in Portugal [69]. The discrepancy in the effects of calcium on body weight/fat loss might result from the different subjects, calcium intake amounts, calcium sources, and calcium intake periods. Thus, due to the various influencing factors, the anti-obesity effects of dietary calcium need to be further studied in different subjects.

## 3. Possible Mechanisms for Calcium’s Anti-Obesity Effects

### 3.1. Effects of Calcium on Adipogenesis

Adipogenesis includes the commitment of mesenchymal stem cells (MSCs) to the adipocyte lineage (preadipocytes) and the terminal differentiation of preadipocytes to mature adipocytes. It is tightly regulated by various signaling molecules and several key adipogenic transcription factors, such as PPARγ and C/EBPα [70]. It has been demonstrated that adipogenesis or hyperplasic adipose expansion is linked to anti-obesity and improved metabolic health [70,71,72]. A large body of evidence has demonstrated that calcium is involved in regulating adipogenesis. Jensen et al. found that high [Ca^2+^]_o_ (5 and 10 mM) inhibited the adipogenesis of 3T3-L1 preadipocytes compared to controls (1.8 mM [Ca^2+^]_o_), with decreased expression of PPARγ and C/EBPα [43]. Similarly, it has been reported that increased [Ca^2+^]_i_ with RyR channel excitomotor caffeine significantly suppresses adipogenesis of mice preadipocytes, with decreased lipid content and *PPARγ* expression [44]. However, Shi et al. demonstrated that increasing [Ca^2+^]_i_ appeared to exert a biphasic regulatory effect on human adipocyte differentiation, inhibiting the early stages while promoting the late stage of differentiation and lipid filling [46]. We also found that high [Ca^2+^]_o_ (4 mM) stimulated adipogenesis of porcine bone marrow MSCs (pBMSCs) by increasing the [Ca^2+^]_i_ level and activating CaMKII and PI3K/Akt-FoxO1 pathways [47]. We further determined that the promotive effects of [Ca^2+^]_o_ on pBMSCs occurred mainly in the commitment phase but not in the terminal differentiation phase (unpublished data). In line with our results, it has been indicated that high [Ca^2+^]_o_ enhances adipogenic differentiation of mice BMSCs [73,74] and stimulates adipogenesis of porcine synovium-derived MSCs [75]. These findings imply that calcium stimulates the early stage (commitment stage) and suppresses the late stage (terminal differentiation stage) of adipogenesis. Taken together, calcium may elicit inhibitory or stimulatory effects on adipogenesis in vitro depending on the calcium concentration, cell types, and culture systems.

Compared with the in vitro findings, the in vivo data may better reflect the role of calcium in adipogenesis. In agreement with the enhanced adipogenesis in pBMSCs, we found that calcium supplementation stimulated adipogenesis in mice fed with HFD, with increased adipocyte number and PPARγ expression in inguinal subcutaneous white adipose tissue [47]. Similarly, Zhang et al. found that calcium propionate supplementation in the diet of Wagyu steers could trigger upregulation of *PPARγ* and *CEBPα* mRNA expression levels, which could cause long-term activation of adipogenesis [76]. Our unpublished study also demonstrated that dietary supplementation of 1% calcium propionate significantly increased expression of adipogenesis marker genes, such as PPARγ and CEBP/α, in the backfat of finishing pigs. It should be noted that while enhanced adipogenesis in vitro is always accompanied by elevated lipid content, increased adipogenesis (or adipocyte number) in vivo does not mean more fat deposition. In fact, we found that the adipocyte diameter/size in calcium-supplemented mice was much smaller than that of HFD-fed mice. As a result, the WAT index, body fat content, and body weight were significantly reduced by calcium supplementation [47]. In agreement with this, dietary calcium supplementation (10 g/kg) significantly inhibited adipocytes hypertrophy, with a remarkable decrease in adipocyte area in VAT of rats [50]. Similarly, dietary calcium supplementation significantly decreased the lipid droplet sectional area in rat BAT [52]. Therefore, dietary calcium can not only stimulate adipogenesis (or hyperplasia) but also inhibit adipocyte hypertrophy (adipocyte size) in vivo.

### 3.2. Effects of Calcium on Fat Metabolism

Fat metabolism in adipocyte involving the synthesis and degradation of fat (or triglyceride, TG) contributes to the hypertrophy (increase in size) and atrophy (decrease in size) of adipocytes, respectively [42]. Therefore, suppression of fat synthesis and/or promotion of fat breakdown will result in smaller adipocytes and thus less fat deposition. It has been implicated that an increase in dietary calcium intake attenuates diet-induced adiposity by modulating adipocyte intracellular Ca^2+^ and thereby coordinately inhibiting lipogenesis and accelerating lipolysis [77]. Sun et al. reported that high [Ca^2+^]_o_ or [Ca^2+^]_i_ leads to reduced intracellular lipid content and decreased expression of lipogenesis genes, such as *FAS* and *LPL*, and increased expression of lipolysis gene *HSL* [44,45]. Meanwhile, the store-operated Ca^2+^ entry (SOCE) induced the phosphorylation of HSL and increased the pHSL/HSL ratio by the activation of cAMP-PKA pathway in 3T3-L1 cells [78]. Consistent with this, dietary supplement with calcium had a protective effect against HFD-induced obesity in mice by enhancing the expression of *HSL* [45]. In addition, it was reported that high calcium diet significantly decreased the FAS activity and triglyceride level and increased lipolytic activity with an elevated level of glycerol content in adipose tissue of male Wistar rats, thus leading to lower adiposity index [79]. Furthermore, dietary calcium supplementation during maternal pregnancy and lactation decreased the mRNA expression of FAS and SREBP-1c in the adipose tissue of adult female offspring [80]. Collectively, calcium may elicit its anti-obesity role by modulating fat metabolism, with decreased fat synthesis and increased fat breakdown.

### 3.3. Effects of Calcium on Adipocyte (Precursor) Proliferation and Apoptosis

It has been demonstrated that calcium is involved in regulating proliferation of preadipocytes or MSCs. We observed that the enhanced proliferation of pBMSCs induced by high extracellular calcium was associated with the activation of the calcium-sensing receptor (CaSR) and ERK signaling pathway [32]. Similarly, Rocha et al. found that activation of CaSR elevated proliferation of LS14 preadipocytes [81]. In addition, the pro-proliferation effects of [Ca^2+^]_o_ have been reported in rat bone marrow-derived progenitor cells [82] and porcine synovium-derived mesenchymal stromal cells [75]. It should be noted that different species, cell types, and/or culture conditions (e.g., calcium concentrations) will cause different proliferative effects of calcium. We found that [Ca^2+^]_o_ promoted pBMSCs proliferation when [Ca^2+^]_o_ was greater than or equal to 4 mM [32]. In contrast, Liu et al. reported that the optimal [Ca^2+^]_o_ for rabbit BMSCs to proliferate was 1.8 mM and that a higher level of [Ca^2+^]_o_ did not change cell proliferation [83]. In addition, it was shown that low calcium (0.09 mM) greatly enhanced the growth rate and extended the lifespan of human adipose-derived MSCs [84].

Regulation of the adipocyte number by stimulating apoptotic cell death is emerging as a potential strategy for prevention and treatment of obesity. Calcium has been implicated to be linked with apoptosis [85]. It has been shown that a sustained increase in intracellular Ca^2+^ triggers apoptotic cell death and that Ca^2+^-mediated apoptosis can be induced in mature adipocytes [86]. Consistent with this, it was reported that high vitamin D and calcium intake activated the Ca^2+^-mediated apoptotic pathway in the adipose tissue of diet-induced obese mice, thus leading to reduced adiposity [49]. In addition, it was shown that calcium caused apoptosis in undifferentiated human adipose tissue-derived MSCs [80]. In contrast, Jensen et al. found that treatment of 3T3-L1 cells with high [Ca^2+^]_o_ did not significantly affect cell number or viability and did not trigger apoptosis [43]. The distinct effects of calcium on apoptosis in MSCs and 3T3-L1 might be due to the different cell types, culture systems, and calcium concentrations. Besides that, promoting the autophagy of adipocyte cells might also play a part in anti-obesity. The relationship between calcium signals and autophagy was reviewed by Bootman et al. [87]. In this review, they summarized that Calcium (Ca^2+^) and Ca^2+^ channels have been shown to control various stages of autophagic flux. In addition, activation of calcium-sensing receptor (CaSR) induced autophagy in LS14 and SW872 preadipocyte cell lines as well as primary human preadipocytes [88]. Taken together, calcium-induced apoptosis of adipocyte (precursor) might contribute to the beneficial effects of calcium on body weight/fat loss.

### 3.4. Effects of Calcium on Thermogenesis

One of the possible mechanisms by which calcium decreases body fat is enhancing thermogenesis/energy expenditure. Calcium has been implicated to be involved in the regulation of energy balance [89]. It has been well documented that brown adipocytes and beige/brite adipocytes are enriched with uncoupling protein 1 (UCP1) and contributors to thermogenesis/heat production and are thus beneficial for body fat loss [90,91,92]. Conceicao et al. reported that dietary calcium supplementation was able to improve BAT thermogenesis capacity in adult rats that were overfed earlier during lactation [52]. In line with the result, our findings demonstrated that calcium supplementation significantly increased BAT thermogenesis in HFD-fed mice, with higher temperature of interscapular BAT (iBAT) and elevated expression of thermogenesis related genes, such as UCP1 and peroxisome proliferator-activated receptor coactivator 1α (PGC1-α) in iBAT [93]. Accordingly, the induction of thermogenic genes in response to β-adrenergic receptor stimulation was suppressed by reduced intracellular calcium in the brown adipocytes of wild-type mice [94]. In contrast, increased intracellular calcium via transient receptor potential vanilloid 2 (TRPV2) facilitated UCP1 expression and heat production [95]. It has been implicated that the calcium-promoted thermogenic capacity of brown adipocytes may be attributed to the increased mitochondrial fusion and mitochondrial–endoplasmic reticulum contacts induced by calcium [96].

However, Parra et al. found that dairy calcium (12 g/kg diet) intake had no effects on *UCP1* expression in BAT and *UCP2* expression in WAT of mice and suggested that activation of thermogenesis is not involved [97]. In addition, it was reported that calcium solely after the induction phase of differentiation specifically suppressed gene expression of *UCP1, PR domain zinc-finger protein 16 (PRDM16), and PGC1-α* [34]. The inconsistent effect of calcium on BAT activation or thermogenesis might be due to the various animal/cell models, calcium doses, and calcium intake durations. Among these factors, calcium dose might be the most important one contributing to the variability.

With regard to calcium and WAT browning, it has been shown that sarco/endoplasmic reticulum Ca^2+^-ATPase 2b (SERCA2b)-mediated calcium cycling can regulate thermogenesis in beige adipocytes [98]. We also found that calcium supplementation in drinking water was able to boost WAT browning, with significantly elevated expression of thermogenesis related genes, including UCP1, PRDM16, and PGC1-α [93]. Taken together, the current evidence suggests that calcium is involved in enhancing thermogenesis by stimulating BAT activation and WAT browning.

### 3.5. Effects of Calcium on Fat Absorption and Fecal Fat Excretion

Decreased fat absorption and increased fecal fat excretion constitute a primary determinant accounting for prevention or treatment of obesity. In animals, it has been demonstrated that high calcium intake depresses fat digestion and absorption in veal calves [99,100]. In addition, at very low concentrations of calcium, the hamster jejunum produced very few chylomicrons, suggesting reduced fat absorption [101]. Furthermore, it has been reported that a high-calcium (2.4%) diet increases fecal excretion of dietary lipid, which might partly contribute to the reduced body fat content in rats [102]. Moreover, Ayala-Bribiesca et al. found that cheddar-type cheeses enriched with calcium led to more abundant calcium soaps, a quantitative index for fecal fatty acids, in rat feces, suggesting elevated fecal fat excretion [103].

In humans, it has been shown that supplementation of calcium decreases fat absorption and increases the fecal excretion of insoluble calcium soaps with fatty acids [104]. Similarly, a short-term increase in dietary calcium intake promoted fecal fat and energy excretion [105]. Increasing calcium intake from low-fat dairy products by 1600 mg/day for seven days doubled the total fat excretion, with no effect on the excretion of bile acids [106]. Christensen et al. estimated that increasing the dairy calcium intake by 1241 mg/day resulted in an increase in fecal fat of 5.2 g/day [107]. In addition, it was reported that supplementation of dairy calcium in conjunction with orlistat augmented fecal fat excretion [108]. Furthermore, oral supplementation of elemental calcium as calcium carbonate dose-dependently increased the percentage of fecal fat secretion to fat intake in men [109]. Moreover, short-term dietary calcium fortification (2200 mg/day total and 550 mg calcium citrate malate) significantly increased dietary saturated fat excreted from 6% to 13% in men [110]. It has been implicated that the formation of insoluble calcium soaps and the alteration of the interfacial organization of hydrolyzed lipids are involved in calcium-induced decreased fat digestion and absorption and increased fecal fat secretion [111,112]. Taken together, the beneficial roles of calcium in decreasing fat absorption and increasing fecal fat excretion might be responsible for its anti-obesity effects.

### 3.6. Effects of Calcium on Gut Microbiota

Emerging evidence has been highlighting an increasingly more important role of gut microbiota in the regulation of obesity [113,114,115,116,117]. It has been indicated that phylum-level changes in gut microbiota composition, decrease in bacterial diversity, and alterations of functional genes and metabolic activities are associated with obesity [118,119,120]. Thus, dietary intervention or modulation of the gut microbiota has the potential to prevent or treat obesity and obesity-related metabolic diseases [121,122,123].

It has been demonstrated that high-calcium diets appear to positively affect gut microbiota composition, favoring the growth of lactobacilli [124]. Similarly, Chaplin et al. showed that calcium supplementation modulates gut microbiota in a prebiotic manner, promoting a healthier metabolic profile, in dietary obese mice [48]. The authors found that calcium supplementation increased the length of the small intestine and the weight of the cecum and cecum feces. Calcium-fed mice exhibited increased levels of *Bifidobacterium* spp. and *Bacteroides/Prevotella* and decreased levels of *Clostridium coccoides* and *Clostridium leptum* [48]. In line with these results, we found that, compared with HFD-fed mice, supplementation of calcium in drinking water increased the community diversity and specific bacterial abundance in feces [125]. In addition, it has been reported that dietary calcium has a substantial influence on gut microbiota in pigs [126], broilers [127], laying hens [128], and white shrimp [129]. To date, the effects of dietary calcium on human gut microbiota remain largely unknown and need to be further explored. Nevertheless, the current evidence in animals suggests that dietary calcium might interfere with gut microbiota, which partly explains the beneficial effects of calcium on body weight/fat loss.

## 4. Conclusions

**Figure 1:**
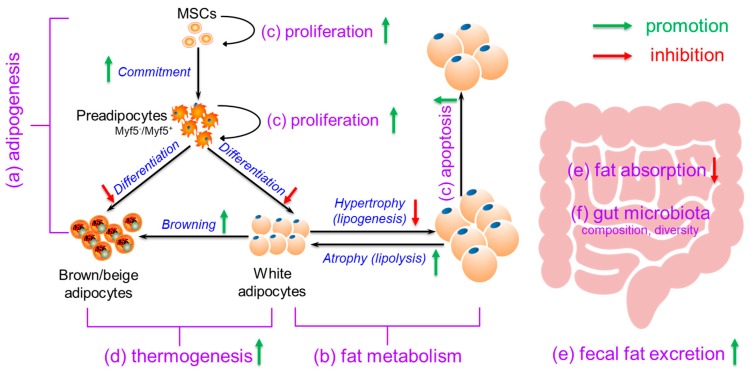
The possible mechanisms for the anti-obesity effects of dietary calcium. Calcium may elicit anti-obesity effects through (a) regulation of adipogenesis, with stimulation on mesenchymal stem cells (MSCs) (or commitment stage) and inhibition on preadipocytes (or differentiation stage); (b) modulation of fat metabolism, with decreased fat synthesis (lipogenesis) and increased fat breakdown (lipolysis); (c) promotion of adipocyte (precursor) proliferation and apoptosis; (d) enhancement of thermogenesis, with increased brown adipose tissue (BAT) activation and white adipose tissue (WAT) browning; (e) suppression of fat absorption and promotion of fecal fat excretion; and (f) modification of gut microbiota composition and diversity.

In this review, we compiled the evidence for the anti-obesity effects of calcium in cell models, animals, and humans. In addition, we summarized the possible anti-obesity mechanisms of calcium, including (a) regulation of adipogenesis, with stimulation on MSCs (or commitment stage) and inhibition on preadipocytes (or differentiation stage); (b) modulation of fat metabolism, with decreased fat synthesis (lipogenesis) and increased fat breakdown (lipolysis); (c) promotion of adipocyte (precursor) proliferation and/or apoptosis; (d) enhancement of thermogenesis, with increased BAT activation and WAT browning; (e) suppression of fat absorption and promotion of fecal fat excretion; and (f) modification of gut microbiota composition and diversity (Figure 1). In conclusion, the current evidence demonstrates the anti-obesity effects of calcium and suggests the potential application of dietary calcium supplementation for prevention or treatment of obesity.

## Abbreviations

BAT	brown adipose tissue
iBAT	interscapular brown adipose tissue
[Ca^2+^]_i_	intracellular calcium
[Ca^2+^]_o_	extracellular calcium
CaSR	calcium-sensing receptor
C/EBPα	CCAAT/enhancer binding protein α
FAS	fatty acid synthetase
HSL	hormone sensitive lipase
LPL	lipoprotein lipase
MSCs	mesenchymal stem cells
pBMSCs	porcine bone marrow mesenchymal stem cells
PGC1-α	peroxisome proliferator-activated receptor coactivator 1α
PPARγ	peroxisome proliferator activated receptor γ
PRDM16	PR domain zinc-finger protein 16
T2DM	type 2 diabetes mellitus
TG	Triglyceride
TRPV2	transient receptor potential vanilloid 2
UCP1	uncoupling protein 1
VAT	visceral adipose tissue
WAT	white adipose tissue
eWAT	epididymal white adipose tissue
iWAT	inguinal white adipose tissue
